# Wastewaters, with or without Hospital Contribution, Harbour MDR, Carbapenemase-Producing, but Not Hypervirulent *Klebsiella pneumoniae*

**DOI:** 10.3390/antibiotics10040361

**Published:** 2021-03-29

**Authors:** Adela Teban-Man, Anca Farkas, Andreea Baricz, Adriana Hegedus, Edina Szekeres, Marcel Pârvu, Cristian Coman

**Affiliations:** 1 NIRDBS, Institute of Biological Research Cluj-Napoca 400015, Romania; adela.teban@icbcluj.ro (A.T.-M.); andreea.baricz@icbcluj.ro (A.B.); adriana.bica@icbcluj.ro (A.H.); edina.szekeres@icbcluj.ro (E.S.); 2 Department of Taxonomy and Ecology, Faculty of Biology and Geology, Babeș-Bolyai University, Cluj-Napoca 400084, Romania; marcel.parvu@ubbcluj.ro; 3 Department of Molecular Biology and Biotechnology, Faculty of Biology and Geology, Babeș-Bolyai University, Cluj-Napoca 400084, Romania; ancuta.farkas@ubbcluj.ro

**Keywords:** *Klebsiella pneumoniae*, wastewaters, hospital input, carbapenemase

## Abstract

Carbapenemase-producing *Klebsiella pneumoniae* (CPKP) isolated from influent (I) and effluent (E) of two wastewater treatment plants, with (S1) or without (S2) hospital contribution, were investigated. The strains belonged to the Kp1 phylogroup, their highest frequency being observed in S1, followed by S2. The phenotypic and genotypic hypervirulence tests were negative for all the strains tested. At least one carbapenemase gene (CRG), belonging to the *bla*KPC, *bla*OXA-48, *bla*NDM and *bla*VIM families, was observed in 63% of CPKP, and more than half co-harboured two to four CRGs, in different combinations. Only five CRG variants were observed, regardless of wastewater type: *bla*KPC-2, *bla*NDM-1, *bla*NDM-6, *bla*VIM-2, and *bla*OXA-48. Sequence types ST258, ST101 and ST744 were common for both S1 and S2, while ST147, ST525 and ST2502 were found only in S1 and ST418 only in S2. The strains tested were multi-drug resistant (MDR), all being resistant to beta-lactams, cephalosporins, carbapenems, monobactams and fluoroquinolones, followed by various resistance profiles to aminoglycosides, trimethoprim-sulphamethoxazole, tigecycline, chloramphenicol and tetracycline. After principal component analysis, the isolates in S1 and S2 groups did not cluster independently, confirming that the antibiotic susceptibility patterns and gene-type profiles were both similar in the *K. pneumoniae* investigated, regardless of hospital contribution to the wastewater type.

## 1. Introduction

Antibiotics are one of the most significant discoveries in microbiology, saving millions of lives by preventing and treating bacterial infections. Since their discovery, they have been used on a large scale in medicine, agriculture, growth promotion and to prevent diseases in animals. Eventually, this facilitated the progress of an antimicrobial resistance (AMR) phenomenon, which rapidly became a growing health safety issue worldwide [1]. Nowadays, AMR is constantly expanding, leading to infections that are more difficult to treat and to an increase in the associated mortality rate [2]. Over time, antibiotic resistance has been recognized for almost every class of antibiotic including carbapenems, which are often called “last line agents” due to their efficiency in treating serious, life-threatening infections caused by Gram-negative and Gram-positive bacteria [3]. Enzyme-mediated carbapenem resistance is the most important clinically, due to the ability of beta-lactamases to inactivate carbapenems, together with other beta-lactam antibiotics [4]. These enzymes fall under three Ambler classes, as follows: class A, with *Klebsiella pneumoniae* carbapenemase (KPC) being the most representative enzyme; class B, known as metallo-β-lactamases (MBLs), having Verona integron-encoded MBL (VIM), and New Delhi MBL (NDM) as most clinically relevant enzymes, and class D, represented by the OXA-type β-lactamases [5]. Moreover, carbapenemases are encoded by genes that are usually found on mobile genetic elements, thus increasing the potential of transmission to other bacteria.

The World Health Organization [2] published a global priority list of antibiotic-resistant bacteria (ARB) that pose the greatest risk to human health. One of the main ARB groups associated with serious and often deadly human infections is the carbapenem-resistant *Klebsiella pneumoniae* complex, composed of non-motile, encapsulated, Gram-negative bacteria, belonging to the Enterobacteriaceae family [6]. *K. pneumoniae* sensu lato is classified into seven phylogroups (Kp1–Kp7), with similar species [7]: Kp1-*K. pneumoniae* sensu stricto (*K. pneumoniae* subsp. *pneumoniae*, *K. pneumoniae* subsp. *rhinoscleromatis*, *K. pneumoniae* subsp. *ozaenae*); Kp2-*K. quasipneumoniae* subsp. *quasipneumoniae*; Kp3-*K. variicola* subsp. *variicola*; Kp4-*K. quasipneumoniae* subsp. *similipneumoniae*; Kp5-*K. variicola* subsp. *tropica*; Kp6-*K. quasivariicola* and Kp7-*K. africana*, [7,8].

The dissemination of antibiotic resistance genes (ARGs) worldwide, especially the carbapenemase family (CRG), became an urgent global health threat [9,10], mostly because their presence was detected in both clinical and environmental settings [11]. Wastewaters represent a hotspot for the development and dissemination of AMR and ARGs [12], being a key factor in this process [13]. Clinical isolates reaching wastewaters contribute to spreading ARGs to non-pathogenic bacteria, leading to new generations of ARBs with different and more aggressive resistance patterns [14,15]. Recent studies [16,17] reported the presence of multi-drug resistant (MDR) *Klebsiella pneumoniae* in different aquatic environments such as rivers, hospital effluents, and post-hospital wastewater. It was suggested that wastewaters are important reservoirs of AMR that contribute to the dissemination of ARBs and ARGs in the environment and from there to the general population [17,18], calling for an increased attention to a possible correlation between clinical isolates and those found in wastewaters or even in surface waters [19,20]. Also, even though hypervirulent *K. pneumoniae* clones (HvKp) are frequently encountered among clinical isolates and a convergence of MDR and virulence factors can occur, there is little information on the prevalence of MDR HvKp in non-hospital sources. To the best of our knowledge, no comparative analysis has been performed among wastewaters that do or do not receive hospital input regarding carbapenem resistance and virulence factors in Gram-negative bacteria. Thus, the purpose of this study was to assess the molecular epidemiology of carbapenemase-producing *Klebsiella pneumoniae* isolated from the influent and effluent of two wastewater treatment plants (WWTPs) with and without hospital contribution.

## 2. Results and Discussion

### 2.1. Carbapenemase Diversity and Hypervirulence of Klebsiella Pneumoniae in the Investigated Wastewaters

**Figure 1:**
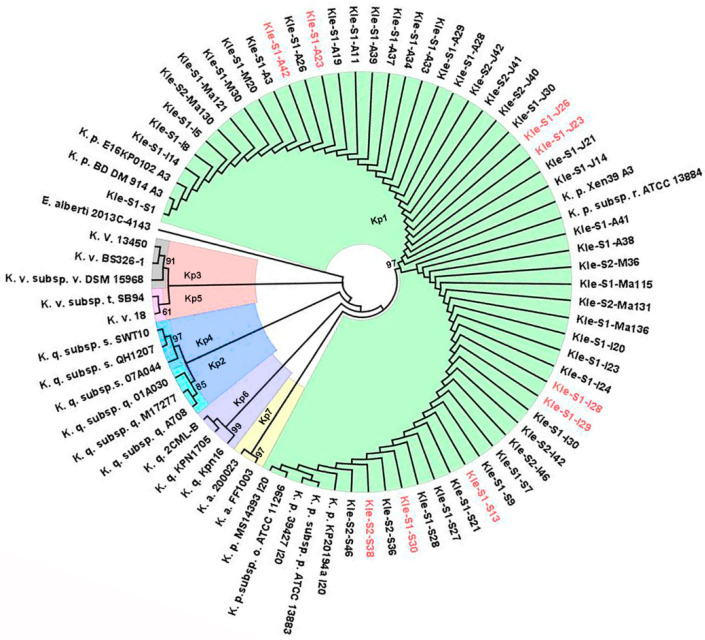
Neighbor Joining tree built on partial *dna*J sequences. Bootstrap values (>60%) are shown adjacent to each *Klebsiella* phylogroup. The annotations of the GenBank taxa were abbreviated as follows: *K. pneumoniae* (K. p.), *K. pneumoniae subsp. pneumoniae* (K. p. subsp. p), *K. pneumoniae subsp. ozaenae* (K. p. subsp. o), *K. pneumoniae subsp. rhinoscleromatis* (K. p. subsp. r), *K. quasipneumoniae subsp. quasipneumoniae* (K. q. subsp. q), *K. quasipneumoniae subsp. similipneumoniae* (K. q. subsp. s), *K. variicola* (K. v.), *K. variicola subsp. variicola* (K. v. subsp. v.), *K. variicola subsp. tropica* (K. v. subsp. t.), *K. quasivariicola* (K. q.) and *K. africana* (K. a.). The codes for the isolates from this study represent: Kle—*Klebsiella* genus; S1, S2—wastewater treatment plants (WWTPs); Ma—March; M—May; I—June; J—July; A—August; S—September. Black font—influent; red font—effluent.

In the present study, 74 presumptive carbapenemase-producing *K. pneumoniae* strains were isolated from the influent (I) and effluent (E) of two wastewater treatment plants over a period of six months: S1—received input from multiple hospitals; S2—had no hospital input. A total of 52 isolates (70%) showed positive carbapenemase activity after the mCIM test, 41 (79%) from I and 11 (21%) from E. The highest number of strains with carbapenemase activity were isolated from S1 (41/79%; I: 32/62%, E: 9/17%) and S2 (11/21%; I: 9/17%, E: 2/4%). After molecular fingerprinting using partial *dna*J gene fragments, the topology of the neighbor-joining method (NJ) tree pointed out seven clusters which overlapped the seven phylogroups of *Klebsiella* with high bootstrap values (Figure 1). The 52 sequences derived from this study fell into the cluster of *Klebsiella pneumoniae* sensu stricto (Kp1), a common inhabitant of wastewater [12,19]. Discussions regarding carbapenemase-producing *Klebsiella pneumoniae* from clinical settings have dominated research in recent years, but the presence of this important pathogen in the environment has rarely been studied. The existence of carbapenem-resistant Enterobacteriaceae in Romania, primarily *Klebsiella pneumoniae*, was reported previously [21] in clinical isolates, hospital, and urban wastewater [20,22], but only in the South-Eastern part of the country.

The molecular screening by polymerase chain reaction (PCR), targeting the presence of specific CRGs (*bla*KPC, *bla*OXA-48, *bla*NDM and *bla*VIM), showed that 33 (63%) out of the 52 isolates possess at least one CRG (Table 1, Supplementary Table S1A), while 19 (37%) most likely have different carbapenem hydrolysis determinants (Supplementary Table S1A). Overall, 15 isolates carried only one CRG, the most frequent being Ambler class A (*bla*KPC, 10 isolates), followed by Ambler class D (*bla*OXA-48, four isolates) and subclass B1 metallo-β-lactamases (MBLs) (*bla*NDM, one isolate). The remaining 18 isolates co-harbored two to four CRGs, in different combinations (Table 1).

Samples from station S1, that receives hospital wastewaters, contained all types of investigated CRGs, in both influent and effluent. It is worth mentioning that all four tested CRGs were present in S2 isolates as well, even if no hospital input is associated, with the exception that only *bla*KPC was observed in the S2 effluent (Table 1). Overall, these findings agree with the results presented previously [18], that undertook a comparison between the presence of these genes from clinical isolates and isolates recovered from aquatic environments to determine their transfer routes from clinical settings to surface waters. Of particular concern is the presence of *K. pneumoniae* isolates with two or multiple CRGs (Table 1), present mainly in the influent, but also in effluent samples. WWTP S2, even if it receives only community wastewater, without hospital contribution, had either a single CRG (*bla*KPC, *bla*NDM or *bla*OXA-48) carrying isolates, in both influent and effluent (*bla*KPC) or just influent (*bla*NDM, *bla*OXA-48) or a combination of penicillinases and metallo-β-lactamases (Table 1).

Out of the 52 *K. pneumoniae* Kp1 isolates, 33 that presented at least one of the targeted CRGs were selected for in-depth characterization, i.e., detection of CRG variants, multilocus sequence typing (MLST), and antibiotic susceptibility profiling. Only five CRG variants were observed, regardless of the wastewater type investigated: *bla*KPC-2, *bla*NDM-1, *bla*NDM-6, *bla*VIM-2, and *bla*OXA-48 (Supplementary Table S1B). Almost all variants, except *bla*NDM-6, were observed in hospital and urban wastewaters [23,24]. *bla*KPC-2, initially described as a variant of *bla*KPC-1, but afterward revised as coding for identical enzymes, was observed in all *K. pneumoniae* isolates from this study, in either raw or treated wastewater. This type of class A CRGs hydrolyze extended-spectrum cephalosporins and all carbapenems, KPC-producing isolates being widespread among various taxonomic groups (Enterobacteriaceae, *Pseudomonas aeruginosa, Acinetobacter baumannii*) and geographical locations (USA, Central, and South America, Europe, Asia) [25]. *bla*NDM-1 and its variant *bla*NDM-6 are quite common in Enterobacteriaceae, especially *bla*NDM-1, which was first described in *K. pneumoniae* and *E. coli* isolated from a patient returning to Sweden from India [26]. Both are associated with different types of plasmids, being easily transmitted to other Gram-negative bacteria. Thus, since their discovery they have spread worldwide, *bla*NDM-1 in particular. The difference between *bla*NDM-1 and *bla*NDM-6 consists of a single substitution of alanine to valine at position 233, and this was first reported in India, in an *Escherichia coli* isolate [27]. Also, previous research demonstrates that these substitutions that occur in *bla*NDM variants can increase the hydrolytic activity against carbapenems [28]. A few years later, *bla*NDM-6 was detected in a *Klebsiella pneumoniae* isolate in Iran [29], and recently was found in Europe, in a clinical isolate of *Acinetobacter baumanii* [30]. Moreover, to our knowledge, no study has revealed the presence of *bla*NDM-6 in clinical isolates from Romania, so the existence of this gene variant in wastewater effluents that are discharged in natural environments is worthy of future investigation. The VIM family is composed of integron-associated MBLs. Even though *bla*VIM-2 is a variant of *bla*VIM-1 originally associated with *P. aeruginosa*, it is starting to be reported in the Enterobacteriaceae family as well, including *K. pneumoniae* [31]. *bla*OXA-48 was first described in *K. pneumoniae*, which together with *E. coli* and *E. cloacae*, are the main drivers of worldwide dissemination of this class of CRGs [32].

The phenotypic and genotypic hypervirulence tests were performed on all *Klebsiella* spp. isolates, with negative results for the String test and PCR amplification of specific genes (data not shown).

### 2.2. Klebsiella Pneumoniae Sequence Types

**Figure 2:**
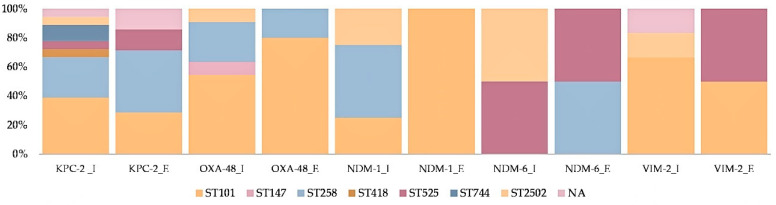
*K. pneumoniae* Sequence Type diversity and the association with carbapenemase genes in raw (influent-I) and treated (effluent-E) wastewaters.

After MLST investigation, seven different sequence types (STs) were identified in the *K. pneumoniae* isolates (Figure 2, Supplementary Table S1B). The most prevalent was ST101 (14 isolates), followed by ST258 (eight isolates), both being observed in raw and treated wastewaters as well. ST525, ST744, ST2502, ST147 and ST418 have lower incidence, the majority being held by one or two isolates only. Treated wastewater samples were composed of isolates belonging to only three distinct STs, i.e., ST101, ST258 and ST525. In addition, a different distribution of STs was observed regarding each tested WWTP: ST258, ST101 and ST744 were common for both S1 and S2 stations, while ST147, ST525 and ST2502 were found only in S1; ST418 appeared only in S2. For three isolates, no ST could be attributed and are currently under investigation for the possible description of novel STs within the *K. pneumoniae* Kp1 phylogroup (data not shown).

ST258, ST101 and ST147 are considered MDR, but not hypervirulent high-risk clones [33,34,35], this being in agreement with our findings regarding the lack of hypervirulence traits. Even though these clones mainly cause severe infections in hospitals, they were common in the wastewaters investigated in this study, regardless of the presence/absence of hospital input. The diversity of *K. pneumoniae* STs and the association with specific CRGs is presented in Figure 2. ST258 is generally associated with *bla*KPC-2 [22]. However, in this study, besides *bla*KPC-2, *K. pneumoniae* isolates within ST258 were associated with other CRGs as well, such as *bla*OXA-48, *bla*NDM-1, *bla*NDM-6 and *bla*VIM-2, in both influent and effluent of S1 and S2. ST101, catalogued as an emerging high-risk clone, was reported frequently in association with *bla*KPC-2, *bla*OXA-48 and *bla*OXA-181 [36], thus agreeing with the results obtained in this study. Moreover, several ST101 isolates from this investigation co-harbored class A, B and D carbapenemases, in raw or treated wastewater from S1. ST147 (*bla*OXA-48) is often associated with clinical and environmental (wastewater and river water) settings [37], but with a lower frequency compared to ST258 and ST101.

Clinical isolates from ST418, ST525 and ST2502 were previously described containing CRGs, such as *bla*KPC-2/3 [38] or *bla*OXA-48 [37]. However, this is the first time they were isolated from wastewaters, with (ST525, ST2502) or without (ST418) hospital contribution. Moreover, *K. pneumoniae* ST2502 isolated in this study, a newly proposed high-risk clone [38], carries all four CRGs investigated (*bla*KPC-2/*bla*OXA-48/*bla*NDM-1/*bla*VIM-2). Besides this, to the best of our knowledge, it is the first time that *K. pneumoniae* ST744 was observed incorporating a CRG (*bla*KPC-2), as this association has not been described previously either in clinical or environmental isolates.

### 2.3. Antimicrobial Susceptibility Profiling and Comparative Analysis among Wastewater Types

**Figure 3:**
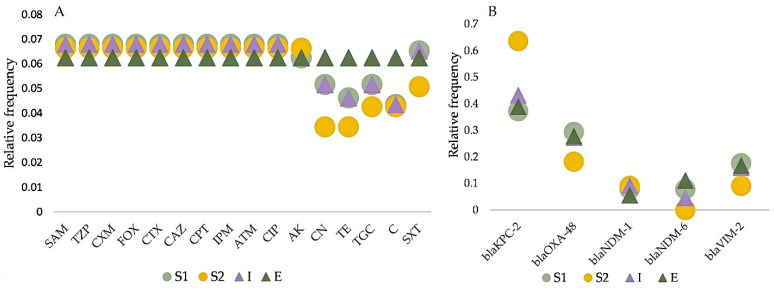
Relative frequency of antibiotic resistance (**A**) and carbapanemase resistance genes (**B**) in *K. pneumoniae* Kp1 isolated from wastewaters. S1 = wastewaters with hospital input; S2 = wastewaters without hospital input; I = wastewater influent; E = wastewater effluent; SAM = ampicillin-sulbactam; TZP = piperacillin-tazobactam; CXM = cefuroxime; FOX = cefoxitin; CTX = cefotaxime; CAZ = ceftazidime; CPT = ceftaroline; IPM = imipenem; ATM = aztreonam; CIP = ciprofloxacin; AK = amikacin; CN = gentamycin; TE = tetracycline; TGC = tigecycline; C = chloramphenicol; SXT = trimethoprim-sulfamethoxazole.

Antimicrobial susceptibility testing revealed that all *K. pneumoniae* isolates described in this study are MDR [39] (Supplementary Table S2). All strains were resistant to beta-lactam antibiotics, including penicillins (ampicillin-sulbactam, piperacillin-tazobactam); cephalosporins (cefuroxime, cefoxitin, cefotaxime, ceftazidime, ceftaroline); carbapenems (imipenem); monobactams (aztreonam) and fluoroquinolones (ciprofloxacin), in both raw and treated wastewaters. A lower percentage of resistance was observed to the following antimicrobials, but only in the case of influent isolates: aminoglycosides—94% (31/33) amikacin and 67% (23/33) gentamicin; 94% (31/33) trimethoprim-sulphamethoxazole; 73% (24/33) tigecycline, 64% (21/33) chloramphenicol and 64% (21/33) tetracycline (Supplementary Table S2). The lowest relative frequencies of resistance were observed with gentamicin and tetracycline in wastewaters without hospital input and with chloramphenicol in isolates from raw wastewaters (Figure 3A). Notably, all the isolates which originate in the effluent of both S1 and S2 WWTPs showed 100% resistance to all the antibiotics tested. These ARBs end up in surface waters, and this could lead to important health issues as some strains of *K. pneumoniae* can act as opportunistic pathogens. Our results are consistent with those of previous studies testing the antimicrobial resistance in *Klebsiella* isolates recovered from wastewaters. They were defined as MDR, being resistant to almost all tested antimicrobial agents [10,17,40]. Besides the resistance to all penicillins, beta-lactamase inhibitors, non-extended spectrum cephalosporins (1st and 2nd generation), extended-spectrum cephalosporins (3rd and 4th generation), broad-spectrum cephalosporins (5th generation), carbapenems, monobactams, fluoroquinolones, we encountered a high percentage (73%) of resistance to tigecycline (TGC). Nowadays, TGC is one of the last resort drugs that can be used for treating the severe infections caused by carbapenem-resistant Enterobacteriaceae, mostly *Klebsiella pneumoniae* [41]. Having a broad spectrum of antimicrobial activity, TGC can overcome the other antibiotic resistance mechanism such as efflux pumps or ribosomal protection used by Enterobacteriaceae against tetracyclines [41]. The results of the present study showed that all the isolates from the treated wastewaters with and without hospital input were resistant to TGC, including important *K. pneumoniae* STs such as ST101, ST258, ST418, ST2502, together with ST744, which was not previously associated with antibiotic resistance. These findings strengthen the previous observations [42] that TGC resistance is growing and soon will become a serious health problem in the population.

Regarding the CRGs, *bla*KPC-2 and *bla*OXA-48 were the most usually found CRGs in *K. pneumoniae* isolated from the two wastewater types, while *bla*NDM-1 and *bla*NDM-6 were the least common (Figure 3B). Comparing the AMR frequencies, no significant differences were observed between S1 and S2 groups. The differences between I and E were statistically significant for tetracycline, chloramphenicol (*p* = 0.012), gentamycin and tigecycline (*p* = 0.0386). Selection of ARB is known to occur during conventional wastewater treatment [13]. Differences between groups were not statistically significant for CRG frequencies (Chi-square test, Fischer’s exact test).

The proportional abundance and diversity of antibiotic resistance in *K. pneumoniae* populations from wastewaters are presented in Table 2. Bacterial isolates displayed increased levels of antibiotic resistance, the average number of antibiotics to which a strain was resistant being 14.55 out of 16 tested. Small variation was observed in the antimicrobial susceptibility of different groups of *K. pneumoniae*. Shannon’s information index (SI) varied between 0 and 0.212. The Gini–Simpson index (GSI) of diversity was between 0.934 and 0.938. The frequency and diversity of resistant phenotypes were very similar in *Klebsiella* spp. isolated from different types of wastewaters. Relying mostly on richness, Shannon’s information index showed a low entropy of AMR profiles, despite the high level of antibiotic resistance. SI was 0 in E due to the lack of elements of surprise, all bacteria from wastewater effluents being resistant to all antibiotics tested. Emphasizing the evenness or balance, the Gini–Simpson index revealed great diversity in AMR profiles in all groups, due to many resistant phenotypes being evenly distributed.

The average number of CRGs per strain was 1.879 with the highest abundance in bacterial strains from S1 (2.04). The highest degree of variation in the carbapenemase-type profiles was observed between S1 (SI = 0.551; GSI = 0.731) and S2 groups (SI = 0.339; GSI = 0.545). When analyzing the carbapenemase resistance profiles between groups, the highest richness and greatest diversity were found in bacteria from wastewater with hospital input, while those originating in the wastewater without hospital input had the lowest gene frequency and diversity. A slight increase in the abundance and diversity of CRG profiles in *K. pneumoniae* was observed after wastewater treatment. Despite the observed variation, the difference between groups was not statistically significant (Wilcoxon signed-rank test).

**Figure 4:**
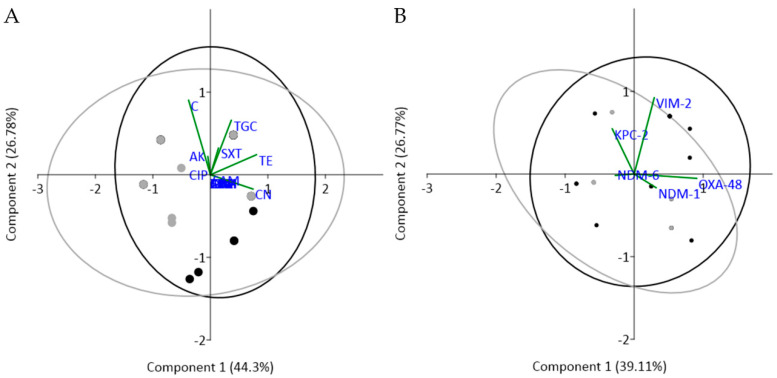
Principal component analysis (PCA) clustering of *K. pneumoniae* isolates according to the susceptibility to 16 antibiotics (**A**) and to the abundance of seven CRGs (**B**). Black dots—S1; gray dots—S2. ATM = aztreonam; AK = amikacin; CN = gentamycin; TE = tetracycline; TGC = tigecycline; C = chloramphenicol; SXT = trimethoprim-sulfamethoxazole.

Principal component analysis (PCA) (Figure 4) showed that the isolates in S1 and S2 groups did not cluster independently, confirming that the antibiotic susceptibility patterns and gene-type profiles were both similar in *K. pneumoniae* isolated from different sources, i.e., similar profiles were observed regardless of hospital contribution to the overall wastewater type. The variation of antibiotic susceptibility was mainly due to the first two components, which contributed with more than 71% of the variance (Figure 4A). The first component correlated with tetracycline (0.657), gentamycin (0.607), and chloramphenicol (–0.310). The second component correlated with chloramphenicol (0.739) and tigecycline (0.540). In the CRG analysis (Figure 4B), the first two components accounted for almost 66% of the variance, where the first component was strongly influenced by *bla*OXA-48 (0.834) and less by *bla*NDM-1 (0.292), while the second one was strongly influenced by *bla*VIM-2 (0.851) and moderately by *bla*KPC-2 (0.502).

### 2.4. Study Limitations

The authors acknowledge the limitations of this study, mainly the reduced number of WWTPs and bacterial isolates targeted. Also, the size (volume of water processed) of each WWTP might have an influence on the number of *K. pneumoniae* isolated. Future studies should consider including several WWTPs of different sizes and degrees of hospital input and, if available, the types and volumes of antimicrobials used in both the hospitals and the community, and test possible correlations among these factors. Overall, our study can be considered a good starting point for implementing a wastewater-based molecular epidemiology survey model to reflect the behaviour of MDR, carbapenemase-producing *Klebsiella pneumoniae* in the community.

## 3. Materials and Methods

### 3.1. Sampling and Strain Isolation

The wastewater samples were collected from the influents and effluents of two different wastewater treatment plants (WWTP) from Cluj County, Romania, during six months in 2020. Station S1 receives hospital input and is designed to process around 115,000 cubic metres (cbm) of wastewater/24h from an average of 400,000 inhabitants. Station S2 has no hospital input, and is currently treating approximately 864 cbm/24h, from around 10,000 inhabitants. Raw and treated 24h-composite wastewater samples were collected using automated refrigerated samplers and transported in a portable cooler to the Environmental Microbiology Laboratory at the Institute of Biological Research Cluj-Napoca, where they were processed on the same day. A volume of 40 mL of raw wastewater and 160 mL of treated wastewater were centrifuged for 20 min at 5000 rpm. After centrifugation, the pellet was resuspended in 1 mL of MacConkey broth. Serial dilutions (10^−1^ to 10^−3^) were performed and a volume of 100 μL was inoculated on 20 mL MacConkey Agar plates, supplemented with 9 μg/mL meropenem (MEM) as per the European Committee on Antimicrobial Susceptibility Testing (EUCAST) guidelines, and incubated overnight at 37 °C. To isolate the presumptive *Klebsiella* spp. strains, only colonies expressing distinct morphological characteristics such as mucoid, lactose fermenting (pink colonies) [43] were selected and inoculated again on MacConkey agar to obtain pure cultures. A total of 77 isolates were selected for further analyses.

### 3.2. Phenotypic Investigation of Carbapenemase Activity and Hypervirulence

The presumptive *Klebsiella* spp. isolates were tested for carbapenemase activity through the modified Carbapenem Inactivation Method (mCIM) [44,45]. A total of 62 isolates showed positive carbapenemase activity and were selected for molecular identification. To detect a phenotypic trait of hypervirulence, the String test method was applied [46,47]. Of note is the mucoviscous phenotype of the AbBAS-1 colonies when grown on agar plates generating a viscous string >5 mm in length between a colony and an inoculation loop (string test), a phenotype that has been associated with hypervirulent *K. pneumoniae* strains [48].

### 3.3. Molecular Identification of Strains

All the presumptive *Klebsiella* spp. isolates were grown overnight at 37 °C on tryptic soy agar (TSA) plates. A 10 μL loop of pure culture was used for DNA extraction using the Quick-DNA Fecal/Soil Microbe Miniprep Kit (ZymoResearch, Irvine, CA, USA), according to the manufacturer’s instructions. The obtained DNA samples were stored at –20 °C and used for molecular analysis.

The *dna*J genetic marker [49] was partially amplified by polymerase chain reaction (PCR), using the specific primers listed in Table 3, and used for taxonomic identification of *Klebsiella* spp. isolates. The PCR assay was performed using the following conditions: initial denaturation at 94 °C for 10 min, followed by 35 cycles of denaturation at 94 °C for 30 s, annealing at 50 °C for 40 s, extension at 72 °C for 50 s and final extension at 72 °C for 5 min [50]. After amplification, the PCR products were verified by electrophoresis in a 1.5% agarose gel and purified using the SAP-Exo Kit (Jena Bioscience GmbH, Thuringia, Germany) by following the provided protocol. The purified amplicons were sequenced by the Sanger method using the service of Macrogen Europe (Amsterdam, Netherlands).

The evolutionary history was inferred using the neighbor-joining method (NJ) [51]. The percentage of replicate trees in which the associated taxa clustered together in the bootstrap test (1000 replicates) is shown next to the branches [52]. The evolutionary distances were computed using the Tamura 3-parameter method [53] which was the best substitution model with the lowest BIC (Bayesian information criterion) score. The rate variation among sites was modelled with a gamma distribution (shape parameter = 0.39). All positions containing gaps and missing data were eliminated (complete deletion option). There were a total of 304 positions in the final dataset. Evolutionary analyses were conducted in MEGA X [54]. The consensus tree was edited with FigTree version 1.4.4 software (Edinburgh, Scotland). The analysis involved 78 nucleotide sequences (52 from the resent study) and 26 from GenBank [55] database. Partial *dna*J gene fragment from *Escherichia albertii* 2013C-4143 (Acc. No. CP030787.2) was used as outgroup. Sequences of *dna*J genes from each representative phylogroup were selected from GenBank as follows: Kp1 (CP040993.1, ACZD01000243.1, CDJH01000053.1, CP054780.1, CP054303.1, CP054268.1, CP052309.1, CP040861.1, CP046949.1), Kp2 (CP030171.1, CP043928.1, CCDF01000065.1), Kp3 (NZ_CP010523.2, CP063898.1, CP026013.1), Kp4 (CBZR010000003.1, SGRH01000005.1, VOIK01000054.1), Kp5 (NZ_CAAHGJ010000005.1, CP048379.1), Kp6 (JADODF010000012.1, CP022823.1, WHZK01000073.1) and Kp7 (CAAHGQ010000001.1, CP059391.1).

**Table 3:** Primer sequences of target genes for species molecular identification, carbapenemase-encoding genes, hypervirulence genes and their amplicon size.

Target Gene	Primer Sequence 5′–3′	Amplicon Size (bp)	Reference
*dna*J	DJF: CNG+GYG+ATYTGTAYGTWCAGGT DJR: T+CRT+CRA+ARAAYTTYTTNACNC	385	[50]
*bla*KPC	KPC Fm: CGTCTAGTTCTGCTGTCTTG KPC Rm: CTTGTCATCCTTGTTAGGCG	232	[56]
*bla*OXA-48	OXA-48 F: GCGTGGTTAAGGATGAACAC OXA-48 R: CATCAAGTTCAACCCAACCG	438	
*bla*NDM	NDM F: GGTTTGGCGATCTGGTTTTC NDM R: CGGAATGGCTCATCACGATC	621	
*bla*VIM	VIM F: GATGGTGTTTGGTCGCATA VIM R: CGAATGCGCAGCACCAG	390	
*iuc*A	F1: AATCAATGGCTATTCCCGCTG R1: CGCTTCACTTCTTTCACTGACAGG	239	[57]
*iro*B	F1: ATCTCATCATCTACCCTCCGCTC R1: GGTTCGCCGTCGTTTTCAA	235	
*peg*-344	F: CTTGAAACTATCCCTCCAGTC R: CCAGCGAAAGAATAACCCC	508	
*_p_rmp*A	F: GAGTAGTTAATAAATCAATAGCAAT R: CAGTAGGCATTGCAGCA	332	
*_p_rmp*A2	F: GTGCAATAAGGATGTTACATTA R: GGATGCCCTCCTCCTG	430	

### 3.4. Molecular Investigation of Hypervirulence Genes and the Diversity of Carbapenemases

Detection of CRGs (*bla*KPC, *bla*OXA-48, *bla*NDM and *bla*VIM) and hypervirulence genes (*iuc*A, *iro*B, *peg*-344, *prmp*A and *prmp*A2) was performed by PCR reaction using the specific primers listed in Table 3 and the following conditions: initial denaturation at 94 °C for 10 min followed by 35 cycles of denaturation at 94 °C for 30 s, annealing at 60 °C for 40 s, extension at 72 °C for 50 s and final extension at 72 °C for 5 min for CRGs and initial denaturation at 95 °C for 2 min followed by 25 cycles of denaturation at 95 °C for 30 s, annealing at 50–59 °C (according to each primer, previously described [57]) for 40 s, extension at 72 °C for 40 s, and final extension at 72 °C for 10 min for hypervirulence genes, respectively. After amplification, the presence of targeted markers was verified by electrophoresis in a 1.5% agarose gel. If a positive amplification was observed, the CRG amplicons were purified using the SAP-Exo Kit (Jena Bioscience GmbH, Thuringia, Germany) as per the manufacturer’s instructions. The purified fragments were sequenced by the Sanger method at Macrogen Europe (Amsterdam, Netherlands). The CRG variants were identified using *blast*X in the National Center for Biotechnology Information (NCBI) database.

### 3.5. Antimicrobial Susceptibility Testing

Antimicrobial susceptibility testing was carried out on selected *Klebsiella* spp. isolates following the Kirby–Bauer disk diffusion method [58]. The selected strains were grown overnight at 37 °C on TSA plates and suspended in 0.85% NaCl sterile solution at a cell density corresponding to 0.5 McFarland standard. Afterwards, 20 mL Muller–Hinton agar plates were inoculated using a sterile cotton swab immersed in the bacterial solution. Antibiotic disks were placed on the inoculated plates, followed by overnight incubation at 37 °C. The standardized approach previously proposed [39] was used, with the following antimicrobials: ampicillin-sulbactam (SAM) (10–10 μg), piperacillin-tazobactam (TZP) (30–6 μg), cefuroxime (CXM) (30 μg), cefoxitin (FOX) (30 μg), cefotaxime (CTX) (5 μg), ceftazidime (CAZ) (10 μg), ceftaroline (CPT) (5 μg), imipenem (IPM) (10 μg), aztreonam (ATM) (30 μg), ciprofloxacin (CIP) (5 μg), amikacin (AK) (30 μg), gentamicin (CN) (10 μg), tetracycline (TE) (30 μg), tigecycline (TGC) (15 μg), chloramphenicol (C) (30 μg), trimethoprim-sulfamethoxazole (SXT) (1,25–23,75 μg). *Escherichia coli* ATCC 25922 was used as a quality-control strain. The inhibition zone was measured in mm and the results were interpreted according to EUCAST [39]. As an exception to EUCAST, the tetracycline and tigecycline inhibition zones were interpreted according to the specifications of the Clinical and Laboratory Standards Institute (CLSI) [59] and the British Society for Antimicrobial Chemotherapy (BSAC) [60], respectively.

### 3.6. Identification of Sequence Types (STs)

A multilocus sequence typing (MLST) assay was performed to characterize the sequence type diversity of *Klebsiella pneumoniae* Kp1 isolates, using the housekeeping genes: *rpo*B, *gap*A, *inf*B, *mdh*, *pgi*, *pho*E, and *ton*B [61]. The genes were amplified by PCR using the primers and protocol described on the *Klebsiella pneumoniae* MLST website [62]. The PCR amplicons were purified using SAP-Exo Kit (Jena Bioscience GmbH, Thuringia, Germany) following the manufacturer’s protocol. After purification, the PCR products were sequenced by the Sanger method using the service of Macrogen Europe, (Amsterdam, Netherlands). The final results were analysed using the database of the Pasteur Institute (Paris, France) and the Public databases for molecular typing and microbial genome diversity, PubMLST [63].

### 3.7. Statistical Analysis

Antimicrobial susceptibility patterns as well as the CRG type results for each isolate were converted to binary matrices (1, 0) and were further analysed for each group. Groups were set as *Klebsiella* spp. from wastewaters with/without hospital input and bacteria from raw/treated wastewater. The variability was assessed in GenAlEx 651b2 (Canberra, Australia) [64] by the proportion of resistant phenotypes/CRGs and Shannon’s information index. Additionally, the weighted Gini–Simpson index of diversity was calculated based on the relative abundance of resistant phenotypes/CRGs. Principal component analysis (PCA) was performed in PAST 3.18 [65] to extract the most informative components that contribute to variation and to explore the clustering of *Klebsiella* isolates. Detection of significant associations between bacterial populations from wastewater with/without hospital input or wastewater inlet/outlet, according to AMR or CRG frequencies, were carried out based on Fisher’s exact test. The significance of differences in diversity indices between groups were analysed using the Wilcoxon signed-rank test. Significance testing (at *p* < 0.05) was performed using Real Statistics Resource Pack software (Release 7.2), copyright (2013–2020), Charles Zaiontz [66].

## 4. Conclusions

The aim of this study was to perform a comparative molecular epidemiology investigation among carbapenemase-producing *Klebsiella pneumoniae* isolated from influent (I) and effluent (E) of two wastewater treatment plants, with (S1) or without (S2) a hospital contribution. From the carbapenem-resistant *Klebsiella* spp. isolates, 70% were carbapenemase-producing (CPKP), belonging to the Kp1 phylogroup based on partial *dna*J sequences. The highest number of CPKP were isolated from S1 (79%; I: 62%, E: 17%), followed by S2 (21%; I: 17%, E: 4%). The phenotypic and genotypic hypervirulence tests were negative for all the strains tested, thus being considered non-hypervirulent. At least one carbapenemase encoding gene (CRG), belonging to the *bla*KPC, *bla*OXA-48, *bla*NDM and *bla*VIM families, was observed in 63% of CPKP, and more than half co-harboured two to four CRGs, in different combinations. Only five CRG variants were observed, regardless of wastewater type: *bla*KPC-2, *bla*NDM-1, *bla*NDM-6, *bla*VIM-2, and *bla*OXA-48. To the best of our knowledge this is the first time that *bla*NDM-6 has been detected in wastewaters, moreover in effluents that are discharged in natural environments. Seven sequence types were observed, some included in the high-risk (ST258, ST101, ST147) or emerging high-risk (ST525 and ST2502) categories, in influent and/or effluent. ST258, ST101 and ST744 were common for both S1 and S2, while ST147, ST525 and ST2502 were found only in S1 and ST418 only in S2. Some of the associations observed in these samples between sequence types and carbapenemase genes have not been described before. The strains tested were MDR, all being resistant to beta-lactams, cephalosporins, carbapenems, monobactams and fluoroquinolones, followed by various resistance profiles to aminoglycosides, trimethoprim-sulphamethoxazole, tigecycline, chloramphenicol and tetracycline. *Klebsiella pneumoniae* from sewage with hospital input carry more abundant and diverse carbapenem resistance genes, compared to isolates from wastewaters without hospital input, but the differences between groups are not statistically significant. Conventional wastewater treatment contributed to the selection of bacterial strains with increased phenotypic resistance but did not affect the genetic profile of carbapenem resistance in *Klebsiella* populations. After principal component analysis, the isolates in S1 and S2 groups did not cluster independently, confirming that the antibiotic susceptibility patterns and gene-type profiles were both similar in the *K. pneumoniae* investigated, regardless of hospital contribution to the wastewater type.

## Figures and Tables

**Table 1:** The distribution of carbapenemase genes (CRGs) in *Klebsiella pneumoniae* isolates.

Combination of CRG Types in the *K. pneumoniae* Isolates	Prevalence of CRG Patterns (*n* = 33)		
	Total	S1_I/S1_E	S2_I/S2_E
*bla*KPC	10	4/1	4/1
*bla*OXA-48	4	2/1	1/0
*bla*NDM	1	1/0	0/0
*bla*VIM	0	0/0	0/0
*bla*KPC/*bla*NDM	2	1/1	0/0
*bla*KPC/*bla*OXA-48	2	1/1	0/0
*bla*KPC/*bla*VIM	2	1/0	1/0
*bla*OXA-48/*bla*NDM	1	1/0	0/0
*bla*OXA-48/*bla*VIM	1	0/1	0/0
*bla*KPC/*bla*OXA-48/*bla*NDM	3	1/1	1/0
*bla*KPC/*bla*OXA-48/blaVIM	5	4/1	0/0
*bla*KPC/*bla*NDM/blaVIM	1	1/0	0/0
*bla*KPC/*bla*OXA-48/*bla*NDM/*bla*VIM	1	1/0	0/0

**Table 2:** Proportional abundance of antibiotic resistance phenotypes and carbapanemase genes in *K. pneumoniae* Kp1 isolated from wastewaters.

Group	N	AMR			CRG		
		*p*	SI	GSI	*p*	SI	GSI
S1	25	14.72	0.177	0.936 (99.88%)	2.04	0.551	0.731 (91.41%)
S2	8	14.00	0.204	0.934 (99.68%)	1.375	0.339	0.545 (68.18%)
I	24	13.44	0.212	0.936 (99.88%)	1.833	0.509	0.704 (87.94%)
E	9	16	0	0.938 (100%)	2.000	0.546	0.728 (91.04%)

N = no. of isolates; *p* = antimicrobial resistance (AMR) or carbapenemase genes (CRG) proportion; SI = Shannon’s information index; GSI = Gini-Simpson index.

## Data Availability

Representative sequences for both taxonomy and CRGs were deposited in GenBank, NCBI, under the accession numbers: MW536488, MW536489, MW527079-MW527085.

## Supplementary Materials

The following are available online at https://www.mdpi.com/article/10.3390/antibiotics10040361/s1, Supplementary Table S1A: Presence/absence of CRGs in *Klebsiella pneumoniae* Kp1 isolates after PCR screening (*n* = 52); Supplementary Table S1B: In-depth characterization of selected *Klebsiella pneumoniae* Kp1 isolates (*n* = 33), Table S2: Antimicrobial susceptibility profiling of *Klebsiella pneumoniae* selected isolates.
